# The Prognostic and Predictive Role of Somatic *BRCA* Mutations in Ovarian Cancer: Results from a Multicenter Cohort Study

**DOI:** 10.3390/diagnostics11030565

**Published:** 2021-03-21

**Authors:** Angela Toss, Claudia Piombino, Elena Tenedini, Alessandra Bologna, Elisa Gasparini, Vittoria Tarantino, Maria Elisabetta Filieri, Luca Cottafavi, Filippo Giovanardi, Stefano Madrigali, Monica Civallero, Luigi Marcheselli, Isabella Marchi, Federica Domati, Marta Venturelli, Elena Barbieri, Giovanni Grandi, Enrico Tagliafico, Laura Cortesi

**Affiliations:** 1 Department of Oncology and Hematology, Azienda Ospedaliero Universitaria di Modena, 41124 Modena, Italy; angela.toss@unimore.it (A.T.); claudia.piombino@outlook.com (C.P.); marchi.isabella@policlinico.mo.it (I.M.); fdomati@unimore.it (F.D.); martaventurelli@msn.com (M.V.); barbieri.elena@aou.mo.it (E.B.); 2 Department of Surgery, Medicine, Dentistry and Morphological Sciences with Transplant Surgery, Oncology and Regenerative Medicine Relevance, University of Modena and Reggio Emilia, 41124 Modena, Italy; monica.civallero@unimore.it; 3 Department of Medical and Surgical Sciences, University of Modena and Reggio Emilia, 41124 Modena, Italy; tenedini.elena@gmail.com (E.T.); enrico.tagliafico@unimore.it (E.T.); 4 Department of Oncology, Arcispedale S. Maria Nuova IRCCS, 42123 Reggio Emilia, Italy; bologna.alessandra@ausl.re.it (A.B.); gaspariniel@ausl.re.it (E.G.); 5 PhD Program in Clinical and Experimental Medicine, University of Modena and Reggio Emilia, 41124 Modena, Italy; vittoriatarantino@hotmail.it; 6 ASL Lecce, Polo Oncologico “Vito Fazzi”, 73100 Lecce, Italy; melisabe.filieri@libero.it; 7 Oncology Unit, Azienda Unità Sanitaria Locale di Modena, Ramazzini Hospital, 41012 Carpi, Italy; lu.cottafavi@ausl.mo.it; 8 Medical Oncology Unit, Azienda Unità Sanitaria Locale, IRCCS di Reggio Emilia, 42122 Reggio Emilia, Italy; giovanardifilippo@gmail.com (F.G.); stefano.madrigali@ausl.re.it (S.M.); 9 Fondazione Italiana Linfomi (FIL), Department of Oncology and Hematology, Azienda Ospedaliero Universitaria di Modena, 41124 Modena, Italy; lmarcheselli@unimore.it; 10 Department of Obstetrics, Gynecology and Pediatrics, Obstetrics and Gynecology Unit, Azienda Ospedaliero Universitaria di Modena, 41124 Modena, Italy; giovanni.grandi@unimore.it; 11 Department of Laboratory Medicine and Pathology, Diagnostic Hematology and Clinical Genomics Unit, Azienda Ospedaliero Universitaria di Modena, 41124 Modena, Italy; 12 Center for Genome Research University of Modena and Reggio Emilia, 41124 Modena, Italy

**Keywords:** BRCA, ovarian cancer, copy number variation, genetic testing

## Abstract

Previous research involving epithelial ovarian cancer patients showed that, compared to germline *BRCA* (gBRCA) mutations, somatic *BRCA* (sBRCA) mutations present a similar positive impact with regard to overall survival (OS) and platinum and PARP (poly (ADP-ribose) polymerase) inhibitor sensitivity. Nevertheless, molecular testing in these studies did not include copy number variation (CNV) analyses of *BRCA* genes. The aim of this study was to explore the prognostic and predictive role of sBRCA mutations as compared to gBRCA mutations in patients who were also tested for CNVs. Among the 158 patients included in the study, 17.09% of patients carried a pathogenic or likely pathogenic gBRCA variant and 15.19% of patients presented pathogenetic or likely pathogenic sBRCA variants and/or CNVs. Overall, 81.6% of the patients included in this study were diagnosed with a serous histotype, and 77.2% were in advanced stages. Among women diagnosed in advanced stages, gBRCA patients showed better progression-free survival and OS as compared to sBRCA and wild-type patients, whereas sBRCA patients did not show any advantage in outcome as compared to wild-type patients. In this study, the introduction of CNV analyses increased the detection rate of sBRCA mutations, and the resulting classification among gBRCA, sBRCA and wild-type patients was able to properly stratify the prognosis of OC patients. Particularly, sBRCA mutation patients failed to show any outcome advantage as compared to wild-type patients.

## 1. Introduction

More than one-fifth of invasive epithelial ovarian cancers present hereditary susceptibility. In about 65–85% of those cases, the genetic defect is a germline that is likely pathogenic or a pathogenic variant in one of the *BRCA* genes [1]. In particular, these germline *BRCA* (gBRCA) variants occur in 4–14% of all women with unselected ovarian cancers (OCs) regardless of family history, in 5–18% of serous OCs, and in about 22% of high-grade serous OC cases [2,3,4,5,6,7,8]. Interestingly, ovarian cancers developed by gBRCA mutation carriers present peculiar clinical and histopathological features: an increased likelihood of a high-grade serous histotype, sensitivity to platinum agents and PARP (poly (ADP-ribose) polymerase) inhibitors, and longer median overall survival (OS) [9,10,11,12,13,14,15]. Besides *BRCA1* and *BRCA2* genes, several other suppressor genes and oncogenes have been associated with hereditary OC, including mismatch repair (MMR) genes, *TP53*, and several genes involved in the double-strand break repair system such as *ATM, CHEK2, RAD51C, RAD51D, BRIP1*, and *PALB2* [1]. *BRCA*-negative tumors with a defect in the homologous recombination system express the BRCAness profile, a specific phenotype with features and behaviors similar to those of *BRCA*-related cancers [16]. In particular, as already shown by several clinical trials, these patients may benefit from treatment with PARP inhibitors. So far, however, validated tests for the identification of BRCAness tumors are still needed [17].

In sporadic OCs, alterations of *BRCA1* and *BRCA2* genes may also occur through somatic mutations or epigenetic silencing. In contrast to germline mutation analysis, somatic *BRCA* testing is routinely performed using next-generation sequencing (NGS), due to the better sensitivity for analyzing tumor tissue DNA as compared to Sanger methodology. In previous studies, somatic *BRCA* (sBRCA) mutations have globally been reported in approximately 5–7% of ovarian cancer cases [17,18,19,20,21,22]. While the prognostic and predictive roles of gBRCA mutations have been largely demonstrated and shared, it is not entirely clear whether harboring a sBRCA mutation brings the same prognostic and predictive advantages. The reasons for this uncertainty are also related to challenges in somatic mutation testing, ranging from the need for high-quality tissue selection and DNA isolation to correct variant interpretation. Moreover, somatic mutations in patients may change over time and according to the site of tumor evaluation, and as a consequence of treatment, cancer evolution, and resistance development [23]. 

A few small cohort studies have previously shown that, like gBRCA, pathogenic sBRCA variants have a similar positive impact on OS as well as platinum and PARP inhibitor sensitivity [3,24,25,26]. On the other hand, a more recent study found no significant impact of sBRCA mutations on progression-free survival (PFS) or OS [27]. Nevertheless, all these studies suffered from relevant shortcomings, particularly in terms of the copy number variation (CNV) analysis of *BRCA* genes.

For the purpose of exploring the prognostic and predictive role of sBRCA mutations as compared to gBRCA mutations and wild-type *BRCA*, clinical pathological characteristics and survival outcomes were evaluated in 158 Italian OC patients. Patients in the study underwent both germline and somatic *BRCA* genetic testing with an NGS amplicon-based approach for tumor samples, specifically designed with shorter amplicons to generate high-quality data for formalin-fixed paraffin-embedded (FFPE) samples.

## 2. Materials and Methods

### 2.1. Study Population and Design

Since 1995, the Modena Family Cancer Clinic (MFCC), which is located in the Emilia Romagna region (Northern Italy), has offered genetic counseling to individuals with a personal or family history of breast cancer (BC) and/or OC in accordance with the criteria recommended by the Emilia Romagna region for *BRCA* genetic testing and the Italian Association of Medical Oncology (AIOM) Guidelines [28,29] (Table 1). The Regional and National indications act on the backdrop of results from Study 19 [30], Study 42 [31], and the SOLO2 studies [32], as well as the Food and Drug Administration (FDA) and the European Medicines Agency (EMA) approval of olaparib for the maintenance treatment of patients with platinum-sensitive relapsed *BRCA*-mutated (germline or somatic) epithelial OC responding to platinum-based chemotherapy. Thus, at the beginning of 2017 the MFCC started to provide somatic and germline *BRCA* genetic testing for patients diagnosed with high-grade serous OC, and in 2018, somatic and germline *BRCA* genetic testing was extended to all patients with non-mucinous and non-borderline epithelial OCs.

During pre-test counseling, information on the family and personal history of cancer is collected, and a family pedigree is drawn up, including third-degree relatives on both the maternal and paternal sides. Healthy women with a family history of BC and/or OC are referred to the MFCC by their general practitioners or the radiologists who perform population-based screening mammography after a preliminary investigation that is required to reach a minimum cut-off point according to specific criteria. On the other hand, BC and OC patients are referred to the MFCC by their oncologists, radiologists, surgeons, or gynecologists. Individuals who meet the criteria can undergo genetic testing; then, in case of a positive result, the search for a specific pathogenic or likely pathogenic variant can be offered to other family members.

For the purpose of our study, the first consecutive OC patients undergoing somatic and germline *BRCA* testing at the MFCC between January 2017 and December 2018 (in accordance with the testing criteria described above) were included into the study regardless of tumor histotype or stage at diagnosis. The patients were referred to the MFCC from five oncology units in the two provinces of Modena and Reggio Emilia. All of them underwent molecular testing at the same laboratory. The patients were then stratified according to whether they were gBRCA mutation carriers (patients carrying a likely pathogenic or pathogenic *BRCA* variant in both somatic and germline samples, with or without other likely pathogenic or pathogenic sBRCA variants), sBRCA mutation carriers (patients carrying a likely pathogenic or pathogenic variant in a tumor sample but not in the germline counterpart), and wild-type patients (wtBRCA, patients carrying no likely pathogenic or pathogenic sBRCA or gBRCA variant). OS, PFS, and time from diagnosis to second disease progression or death from any cause (PFS2) were evaluated in each group. Finally, in order to assess the possible differences in outcome between somatic single nucleotide variants (SNVs)/deletions (indels) and CNVs, sBRCA patients were then divided into two subgroups: patients with somatic SNVs/indels (s1BRCA) and those with somatic CNVs (s2BRCA). Patients with both SNVs/indel and CNVs were included in the s1BRCA group.

### 2.2. BRCA Somatic Testing and Variant Interpretation

Somatic analyses were carried out on formalin-fixed paraffin-embedded (FFPE) specimens from either primary carcinomas or related metastases, prepared following the College of American Pathologist Guidelines [33]. In 40 cases, tissues came from core biopsies, whereas 118 samples were surgical specimens of the primary tumor. Appropriate tissue samples were eventually macrodissected to enrich tumor content, and the DNA was isolated from selected areas with neoplastic cell content >50%. An automated specific DNA extraction protocol was adopted (Maxwell® 16 FFPE Tissue LEV DNA Purification Kit, Promega, Madison, Wisconsin, USA) and fluorometric quantification was undertaken to assess the exact amount of double stranded DNA (dsDNA) (Qubit dsDNA High Sensitivity, Thermo Scientific, Waltham, Massachusetts, USA). All the 158 samples showed a sufficient quantity of extracted DNA to continue the analysis. Amplicon-based library setup and sequencing were performed via a totally automated *BRCA1* and *BRCA2* Oncomine protocol with the IonChef and IONS5 platforms (Thermo Scientific), starting from a minimum of 15 nanograms of DNA per sample. The Oncomine panel covers complete coding sequences and intron–exon junctions of *BRCA1* and *BRCA2* genes (amounting to 265 amplicons and 64-bp exon padding). Sequencing depth was set according to the percentage of tumor cells in the samples under analysis in order to reach a minimum variant coverage of 50 times and detect a minimum variant allele frequency (VAF) as low as 5%.

Data were analyzed for either single nucleotide variants (SNVs), small insertions, or deletions (indels) through a proprietary Ion Reporter analysis workflow with parameters for somatic DNA samples, personalized with a hot-spot file calling for the presence or absence of all of the likely pathogenic or pathogenic *BRCA1* and *BRCA2* variants annotated in Clinvar, LOVD, ENIGMA, and internal private databases at the moment of sequencing. 

Moreover, data were analyzed for CNVs through the Ion Reporter analysis workflow using a proprietary algorithm named the Variability Correction Informatics Baseline (VCIB) that uses an informatics baseline created with 75 diverse samples (with no *BRCA1/2* CNVs) to allow the assessment of corrected log2 ratios of amplicons of identified CNV regions in input sample data. This was followed by a correction for the percent tumor cellularity recorded for the sample to give copy number and confidence interval data for the identified CNV regions. Both algorithms employed to compute the corrected log2 ratios and the correction for the tumor fraction are proprietary.

Moreover, genetic variant annotations were integrated with open-source bioinformatics tools customized and internally validated (Annovar [34] and Variant Effect Predictor [35]). The annotations were then reported using the international standard Human Genome Variation Society (HGVS) nomenclature and classification into five classes according to the American College of Medical Genetics and Genomics (ACMG criteria) [36]. 

Every likely pathogenic (C4) or pathogenic (C5) SNV or indel found via NGS in the somatic sample was confirmed by Sanger sequencing in both somatic and germline DNA patient samples. Germline DNA was isolated with an automated method implemented on the QIAsymphony platform (Qiagen, Germantown, Maryland, USA) starting from 300 μL of peripheral blood samples collected in EDTA tubes; the quantity and quality of nucleic acids were checked using a Qubit dsDNA High Sensitivity kit and Nanodrop (Thermo Scientific). Sanger sequencing was performed with predesigned Invitrogen M13 forward and reverse primers and the BigDye™ Direct Cycle Sequencing Kit, sequenced with the Applied Biosystems® 3500xL Dx Genetic Analyzer platform and analyzed with Minor Variant Finder or SeqScape3 software (ThermoFisher Scientific). 

The CNVs found in the somatic samples could not be confirmed with an alternative method in the somatic samples, but their presence/absence was ascertained in the matched germline sample via the Multiple Ligation Probe Amplification method (MLPA, MRC Holland, Amsterdam, The Netherlands), performed with the Applied Biosystems® 3500xL Dx Genetic platform and analyzed with Coffalyser.Net (MRC Holland) software updated to the latest available version.

### 2.3. Statistical Analysis

Data were analyzed using Fisher’s exact test to identify associations between categorical variables. Two-tailed *p* values of <0.05 were considered statistically significant. OS was measured from the date of diagnosis until death from any cause or date of last known contact for living patients. PFS was defined as the date of diagnosis to progressive disease or death from any cause, whereas PFS2 was defined as the time from diagnosis to second disease progression or death from any cause. The OS, PFS, and PFS2 distributions were calculated using the Kaplan–Meier method and time-to-event distributions were compared using the log-rank test (univariate regression). The Cox regression model was used to estimate the hazard ratio of factors included in multivariable analyses. Statistical analyses were performed using Stata version 14·2 (StataCorp. LLC, College Station, TX, USA), and SPSS v20.0 (IBM Corp., Armonk, NY, USA). 

## 3. Results

### 3.1. Patient Characteristics

Patient characteristics are reported in Table 2. One-hundred-fifty-eight patients were included in the analyses. A total of 27 (17.09%) patients carried a pathogenetic gBRCA mutation: 18 (66.7%) in *BRCA1* and 9 (33.3%) in *BRCA2*. Moreover, 24 (15.19%) patients presented an sBRCA mutation: 10 (41.7%) in *BRCA1*, 13 (54.2%) in *BRCA2*, and 1 (4.2%) in both the *BRCA1* and *BRCA2* genes. Finally, 107 (67.72%) patients presented no mutations. The patients carrying a gBRCA or sBRCA mutation were significantly younger (respectively 58 and 56 years) than the wild-type patients (70 years) (*p* = 0.04). The International Federation of Gynecology and Obstetrics (FIGO) stage at diagnosis was advanced (stage III–IV) in 78.2% of cases, with no significant difference among subgroups (*p* > 0.05). The most frequent histotype was serous for all subgroups (92.6% in gBRCA, 79.2% in sBRCA, 79.4% in wtBRCA) (*p* > 0.05). Although not statistically significant in this sample, gBRCA mutation carriers were more likely to present a BC and/or OC family history, to be diagnosed at advanced stages (85.2%), and to have a serous histotype (92.6%) as compared to sBRCA and wtBRCA patients. While 61.7% of wtBRCA carriers, 59.3% of gBRCA mutation carriers, and 79.2% of sBRCA mutation carriers underwent surgery upfront (*p* > 0.05), 18.5% of gBRCA patients underwent neoadjuvant chemotherapy, as compared to 15.9% of wtBRCA and 0% of sBRCA patients (*p* = 0.05). Besides, 24.7% of wtBRCA patients, 19.2% of gBRCA mutation carriers, and 26.1% of sBRCA mutation carriers presented macroscopic residual disease after surgery (*p* > 0.05). All patients underwent platinum-based first-line treatment, except one of the sBRCA (4.2%) carriers, who did not receive any systemic treatment. 

### 3.2. Outcome Analysis

**Figure 1:**
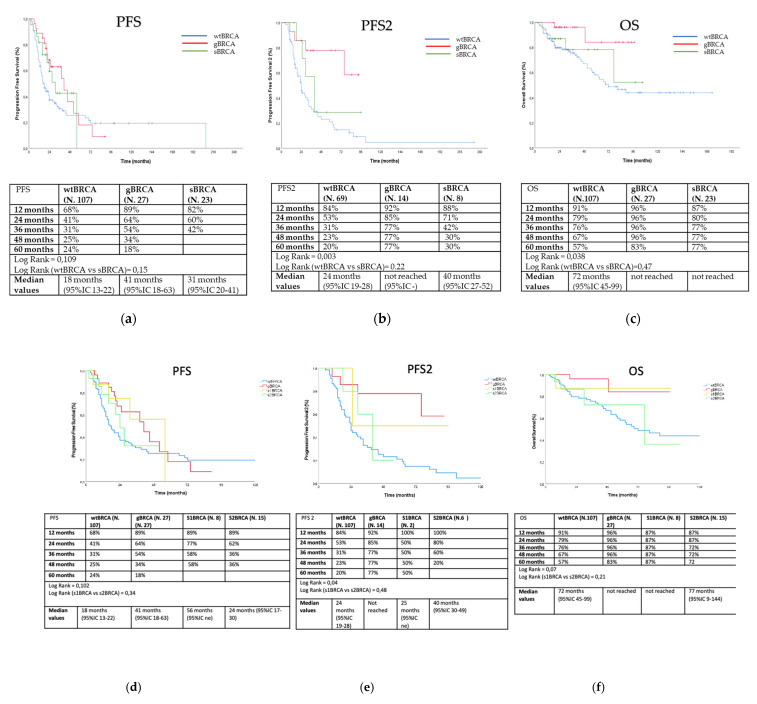
Outcome analyses of PFS (**a**,**d**), PFS2 (**b**,**e**), and OS (**c**,**f**) according to subgroups in the overall population (blue line for wtBRCA, red line for gBRCA, dark green line for sBRCA, yellow for s1BRCA, and light green line for s2BRCA). PFS: progression-free survival; PFS2: progression free survival 2; OS: overall survival; wtBRCA: wild-type *BRCA*; gBRCA: germline *BRCA;* sBRCA: somatic *BRCA*; s1BRCA: somatic SNVs/indels of *BRCA*; s2BRCA: somatic CNVs of *BRCA*.

Outcome analyses were conducted with 157 patients since for one patient the data regarding treatment were not available. Overall, after a median follow-up of 45.7 months, the median PFS was observed to be 18 months in wild-type patients, 41 months in gBRCA carriers, and 31 months sBRCA mutation carriers (log rank = 0.109) (Figure 1a). No patient received PARP inhibitors as maintenance after first-line therapy. Median progression-free survival 2 (PFS2) was 24 months in wild-type patients, not obtained in gBRCA carriers, and was 40 months for sBRCA mutation carriers (log rank = 0.003) (Figure 1b). Three sBRCA (12.5%) and six gBRCA (22.2%) mutation carriers received a PARP inhibitor as maintenance after second-line therapy (eight patients received olaparib and one patient niraparib). Median OS was 72 months in wild-type patients, and not obtained in gBRCA carriers and sBRCA mutation carriers (log rank = 0.038) (Figure 1c). sBRCA carriers did not show any advantage in terms of PFS (log rank = 0.15, Figure 1a), PFS2 (log rank = 0.22, Figure 1b), and OS (log rank = 0.47, Figure 1c) as compared to wild-type patients. Finally, PFS, PFS2, and OS did not significantly differ between s1BRCA and s2BRCA patients (log rank = 0.34, Figure 1d; log rank = 0.48, Figure 1e; log rank = 0.21, Figure 1f). 

**Figure 2:**
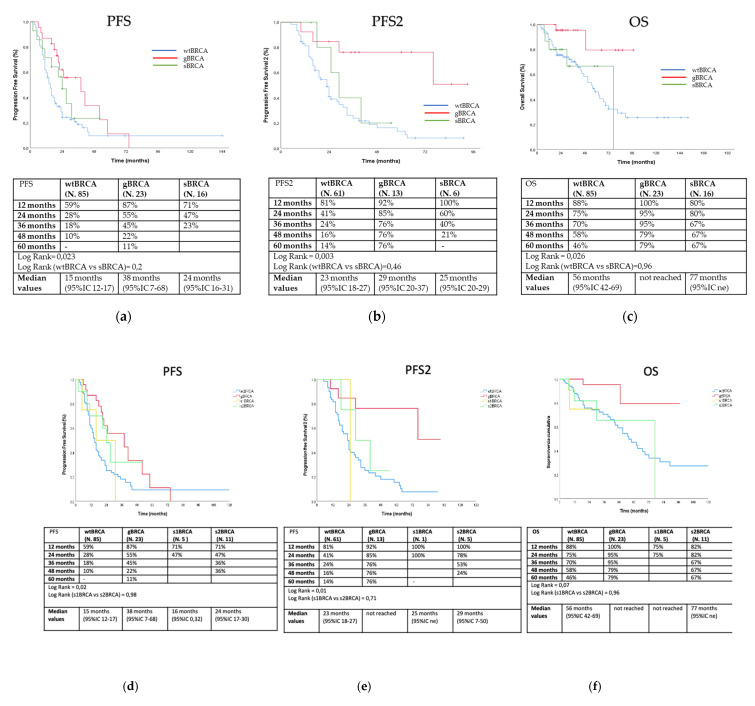
Outcome analyses of PFS (**a**,**d**), PFS2 (**b**,**e**), and OS (**c**,**f**) according to subgroups of patients diagnosed in advanced stages (blue line for wtBRCA, red line for gBRCA, dark green line for sBRCA, yellow for s1BRCA, and light green line for s2BRCA).

Considering only 122 individuals with advanced-stage disease at diagnosis (stage III–IV), after a median follow up of 15 months, median PFS amounted to 15 months in wild-type patients, 38 months in gBRCA carriers, and 24 months in sBRCA mutation carriers (log rank = 0.023) (Figure 2a). Median PFS2 was 23 months in wtBRCA patients, 29 months in gBRCA carriers, and 25 months in sBRCA mutation carriers (log rank = 0.003) (Figure 2b). In this subgroup, two sBRCA (12.5%) and six gBRCA (26.1%) mutation carriers received a PARP inhibitor as maintenance after second-line therapy (seven patients received olaparib and one patient niraparib). Finally, median OS was 56 months in wild-type patients, not obtained in gBRCA carriers, and 77 months in sBRCA mutation carriers (log rank = 0.026) (Figure 2c). In this subgroup as well, sBRCA carriers did not show any advantage in terms of PFS (log rank = 0.2, Figure 2a), PFS2 (log rank = 0.46, Figure 2b), and OS (log rank = 0.96, Figure 2c) as compared to wild-type patients. Finally, PFS, PFS2, and OS did not significantly differ between s1BRCA and s2BRCA patients (log rank = 0.98, Figure 2d; log rank = 0.71, Figure 2e; log rank = 0.96, Figure 2f). 

In the univariate analysis, several clinical features had a significant negative impact on the OS of wtBRCA patients, including age ≥ 50 years (*p* = 0.005), stage III–IV (*p* = 0.04), serous histotype (*p* = 0.03), no surgery (*p* = 0.01), and presence of residual disease (*p* = 0.02). Moreover, age ≥ 50 years (*p* = 0.001), stage III–IV (*p* = 0.001), no surgery (*p* = 0.001), and presence of residual disease (*p* = 0.01) were also predictive of inferior PFS (as shown in Table S1). In the multivariate analysis, FIGO stage, surgery, and residual disease carried significance (*p* < 0.05) for both OS and PFS (Table 3).

### 3.3. Detected BRCA Mutations 

The detected variants in *BRCA1* and *BRCA2* gene are reported in Table 4. 

Twenty-four patients were identified as somatic carriers (sBRCA) (patients 1–24) in either *BRCA1* (12 patients) or *BRCA2* (15 patients). Three patients (number 9, 12, and 23 in Table 4) were reported to have two different sBRCA variants. Fifteen patients presented a somatic CNV, and six patients carried likely pathogenic or pathogenic variants, whereas three patients showed both a likely pathogenic or pathogenic variant and a CNV. 

Of the 27 gBRCA carriers (patients 25–51), two patients also presented another sBRCA mutation in the analyzed tissue (patients 35 and 49). Overall, 18 gBRCA1 and 9 gBRCA2 mutations were detected. Only one patient presented a CNV, which was detected in the somatic analysis and confirmed by MLPA in the germline counterpart.

**Figure 3:**
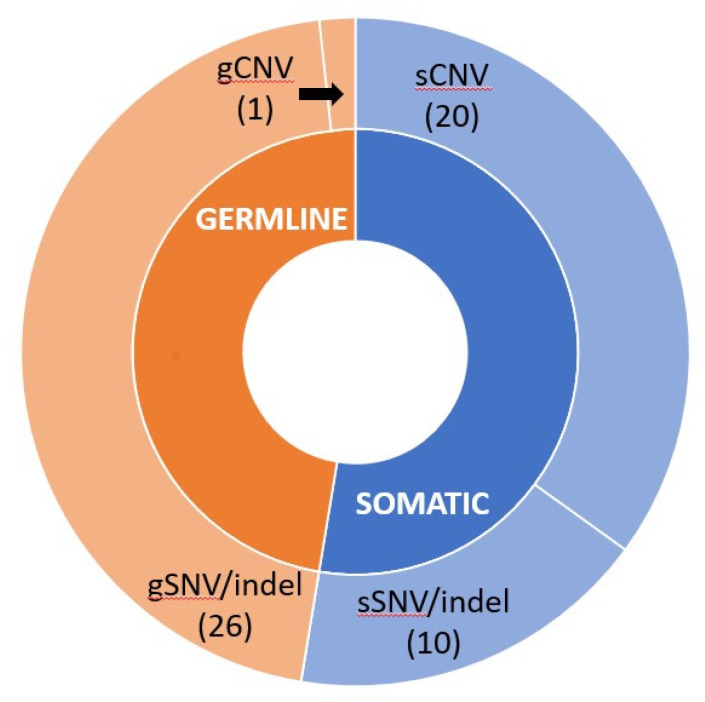
Hierarchical donut chart of likely pathogenic or pathogenic gBRCA and sBRCA variants. The graph shows the origin of mutations found in the inner circle (orange for germline, blue for somatic) and the kinds of variants (CNV or SNV/indel) in the outer circle. Numbers refer to the amount of likely pathogenic or pathogenic variants detected. CNV: copy number variation; SNV: single nucleotide variant.

If germline and somatic carriers seemed to be equally represented in *BRCA*-mutated patients (27 gBRCAs vs. 24 sBRCAs), CNVs seemed to be much more represented in somatic as compared to germline variants (20 sCNVs vs. 1 gCNV). On the other hand, SNVs or indels were more frequently detected as germline variants (26 gSNVs vs. 10 sCNVs), as shown in Figure 3. 

The variant allele frequency (VAF) for all the likely pathogenic or pathogenic variants in the tissues analyzed was >50%, except for in three cases. For these, Sanger sequencing and Minor Variant Finder analysis (see Materials and Methods) confirmed the same frequencies found with NGS in the somatic sample and excluded their presence in their germline counterparts. Low variant frequencies in non-constitutive variants may be due to the molecular heterogeneity of the tumor samples.

## 4. Discussion

The prognostic and predictive role of sBRCA mutations is not entirely clarified. Only a few small cohort studies previously showed that, as compared to gBRCA mutations, sBRCA mutations had a similar positive impact on OS and platinum and PARP inhibitor sensitivity [3,24,25,26]. On the other hand, a recent study found no significant impact of sBRCA mutations on PFS or OS [27]. In all these studies, nonetheless, molecular testing of patients did not include CNV analyses of the two *BRCA* genes. To our knowledge, this is the first study exploring the prognostic and predictive role of sBRCA mutations as compared to gBRCA mutations in patients who were also tested for somatic copy number variations.

The patients were classified according to the presence of sBRCA variants, gBRCA variants, or no pathogenic variants. A total of 17.1% of the patients carried a pathogenetic gBRCA mutation (66.7% *BRCA1* and 33.3% *BRCA2*), while 15.2% were sBRCA carriers (41.7% *BRCA1*, 54.2% *BRCA2,* and 4.2% *BRCA1*+*BRCA2*). Overall, 81.6% of the patients included in this study were diagnosed with a serous histotype, since the MFCC started to provide *BRCA* genetic testing only to patients diagnosed with high-grade serous OC and then to all patients with non-mucinous and non-borderline epithelial OCs. The high rate of serous histotypes could justify the high prevalence of gBRCA mutations detected in our study population. This is in line with data for serous tumors in the literature [2,3,4,5,6,7,8]. Besides, the analysis pipeline on these NGS data is able to detect both SNVs and CNVs, leading therefore to an increase in the detection rate of sBRCA mutations and thus explaining the higher rate of sBRCA mutations in this cohort of OC patients. Without CNVs, sBRCA carriers would represent 6.3%, as previously reported in the literature [17,18,19,20,21,22]. Otherwise, tumors are well known to have increased genomic instability and CNVs [37]. The overrepresentation of somatic CNVs in patients with either *BRCA1* or *BRCA2* mutations is therefore not surprising. Nevertheless, this finding should be further corroborated in larger series of patients, and an alternative confirmatory method for CNV in the somatic samples should be assessed. For this purpose, it could be interesting to confirm *BRCA1* CNVs by using the probe already used for the human epidermal growth factor receptor 2 (HER2) fluorescence in situ hybridization (FISH) test on chromosome 17.

Patients carrying a gBRCA or sBRCA mutation were significantly younger than the wild-type patients. While this finding may not be statistically significant due to the small sample size, gBRCA mutation carriers were predictably more likely to present a family history of BC and/or OC, have a serous histotype, and be in the later stages at diagnosis as compared to the other patients. A higher percentage of gBRCA patients compared to wtBRCA and sBRCA carriers underwent neoadjuvant chemotherapy, possibly due to the higher rate of advanced stage at diagnosis in this group. Most of these gBRCA patients (80.8%) had no residual disease after surgery. On these grounds, several ongoing studies on *BRCA* mutation carriers are evaluating the role of neoadjuvant chemotherapy as compared to up-front surgery in this very chemosensitive subgroup of patients which is frequently diagnosed in the later stages.

Regarding outcomes in the overall population, median PFS did not significantly differ across the three groups, while gBRCA patients showed better PFS2 and OS as compared to sBRCA and wild-type patients. It is likely that the very long PFS of stage I and II OC patients mitigates the biological differences among the three subgroups, whereas the advantages of gBRCA patients emerge in PFS2 and OS after the first relapse, when disease becomes advanced. On the other hand, among women diagnosed in advanced stages (stage III–IV), gBRCA patients showed better PFS, PFS2, and OS compared to all the other patients. The advantage in terms of PFS2 and OS could also be explained by the use of PARP inhibitors as maintenance after the second line of treatment in 26.1% of gBRCA patients as compared to only 12.5% of sBRCA patients and none with wtBRCA. Interestingly, in both the overall population and stage III and IV patients, sBRCA did not show any advantage in terms of PFS, PFS2, and OS as compared to wild-type patients. Overall, our results confirm the recent results of You and colleagues [27] and are apparently in contrast with previous works by Lesnock et al. [24] and Hennessy et al. [3]. Furthermore, no significant differences in PFS, PFS2, and OS were observed between SNVs/indels and CNVs, suggesting that these populations are homogeneous in terms of outcome, while they seem to differ from gBRCA patients. Finally, as expected, in the multivariate analyses, advanced FIGO stage, no surgery, and the presence of residual disease in wtBRCA patients were predictive of poorer outcomes.

Our study presents some limitations that should be underlined. First of all, it should be noted that our current data set is not large enough to enable us to definitively determine whether there is a biological difference between sBRCA- and gBRCA/wtBRCA-mutated tumors and, most of all, between SNVs/indel and CNVs. The small sample size likely explains also the lack of significant results in the univariate and multivariate analyses for the sBRCA and gBRCA groups. Secondly, although the FFPE specimens were prepared following the College of American Pathologist Guidelines [33], poor formalin fixation of samples and formalin itself might have resulted in fragmented DNA and C>U deamination, increasing the risk of false mutated calls. Other limitations are the reliability of existing databases to ascertain pathogenicity in the somatic setting and the temporal and spatial inter- and intra-tumor genetic heterogeneity. In particular, the mutational profile may change over time in the same lesion or may vary in the same individual according to tumor sampling. Indeed, cancer is a dynamic and heterogeneous entity following the principles of clonal evolution, with different areas of the same primary tumor showing different genomic profiles and with metastases acquiring new molecular aberrations as compared to their primary tumors. Additionally, the study lacks gBRCA OC patients undergoing PARP inhibitors as maintenance after first-line therapy and the patients were not evaluated for alterations in other genes involved in the homologous recombination repair system, which could bring survival advantages or platinum/PARP sensitivity like *BRCA* genes [38]. Finally, since the study population included 66 patients diagnosed at least one year before genetic testing, a survival bias should be taken into consideration. Nevertheless, these patients were equally distributed among the three study groups (gBRCA, sBRCA, and wtBRCA), and therefore it is unlikely that this bias could significantly affect the differences in outcome observed.

## 5. Conclusions

In this study the introduction of CNV analyses increased the detection rate of sBRCA mutations, and the resulting classification into gBRCA, sBRCA, and wild-type patients was able to properly stratify OC patient prognosis. In particular, gBRCA mutation carriers presented the best PFS, PFS2, and OS, while sBRCA carriers failed to show any outcome advantage as compared to wild-type patients.

## Figures and Tables

**Table 1:** The Modena Family Cancer Clinic (MFCC) criteria for genetic testing in BC and OC patients. BC: breast Cancer; OC: ovarian cancer.

BC and OC in the Same Patient or Family
OC, fallopian tube, or primary peritoneal cancer (mucinous and borderline types excluded) at any age
Male BC
Triple-negative BC diagnosed at ≤60 years
BC diagnosed at ≤35 years
At least 2 first-degree blood relatives with BC with at least 1 diagnosed ≤40 years or with a bilateral presentation in the same family

**Table 2:** Patient and tumor characteristics by *BRCA* mutational status. wtBRCA: wild-type *BRCA*; gBRCA: germline *BRCA;* sBRCA: somatic *BRCA*; SD: standard deviation; FIGO: International Federation of Gynecology and Obstetrics.

	wtBRCA (*n* = 107, 67.7%)		gBRCA (*n* = 27, 17.1%)		sBRCA (*n* =24, 15.2%)		*p* Value
Median age (SD)	70.50 (0.71)		58.50 (19.09)		56.00 (16.97)		0.04
	n	**%**	n	**%**	n	**%**	
Family history of BC ^1^	38	35.51	14	51.85	9	37.50	>0.05
Family history of OC ^1^	7	6.54	6	22.22	4	16.67	>0.05
Family history of BC+OC ^1^	2	1.87	4	14.81	1	4.17	>0.05
FIGO stage							
I	11	10.28	2	7.41	6	25.00	>0.05
II	12	11.21	1	3.70	2	8.33	
Total I–II	23	21.50	3	11.11	8	33.33	
III	50	46.73	18	66.67	13	54.17	
IV	33	30.84	5	18.52	3	12.50	
Total III–IV	83	77.57	23	85.19	16	66.67	
*Unknown*	1	0.93	1	3.70	0	0	
Histotype							
Serous	85	79.44	25	92.59	19	79.17	>0.05
*Mucinous*	2	1.87	0	0	0	0	
*Clear Cells*	4	3.74	0	0	3	12.50	
*Endometrioid*	16	14.95	1	3.70	2	8.33	
*Transitional*	0	0	1	3.70	0	0	
Non-serous	22	20.56	2	7.41	5	20.83	
Surgery							
No	14	13.08	1	3.70	1	4.17	>0.05
Upfront	66	61.68	16	59.26	19	79.17	
Interval	10	9.35	5	18.52	4	16.67	
Post-neoadjuvant therapy	17	15.89	5	18.52	0	0	0.05
Residual							
yes	23	24.73	5	19.23	6	26.09	>0.05
no	70	75.26	21	80.77	16	69.57	
*Unknown*	0	0	0	0	1	4.35	
First line therapy							
Platinum-based	107	100	27	100	23	95.83	>0.05
Non-platinum-based	0		0		0	0	
*None*	0		0		1	4.17	

^1^ from first to third-degree relatives.

**Table 3:** Multivariate analysis of indices predicting OS and PFS in wtBRCA. HR: hazard ratio; CI: confidence interval.

		OS		PFS	
		wtBRCA		wtBRCA	
Factor	Status	HR (95 CI)	*p*-Value	HR (95 CI)	*p*-Value
Age	<50	1.00		1.00	
	≥50	1.02 (0.78–1.56)	0.9	0.24 (0.09–1.54)	0.83
FIGO	1–2	1.00		1.00	
	3–4	1.69 (1.18–10.8)	0.04	0.17 (0.08–0.61)	0.05
Serous histotype	No	1.00		1.00	
	Yes	0.35 (0.18–1.01)	0.26	1.32 (0.21–4)	0.42
Surgery	Yes	1.00		1.00	
	No	0.43 (0.07–0.99)	0.001	1.34 (1.07–1.92)	0.003
Residual	No	1.00		1.00	
	Yes	0.31 (0.13–0.77)	0.004	1.7 (1.02–2.4)	0.01

**Table 4:** Detected variants in the *BRCA1* and *BRCA2* gene for each patient. VAF: variant allele frequency; CN: copy number.

Patient	*BRCA1*	*BRCA2*	VAF in the Tested Somatic Sample (%)	Copy Number (CN) Call in the Tested Somatic Sample	Confirmed in the Matched Germline Sample	sBRCA/ gBRCA
1	/	/	/	BRCA2DEL entire gene; CN = 1	no	somatic
2	/	c.993_994delAAinsG,p.(Ile332PhefsX17)	52.6		no	somatic
3	c.4675+1G>A	/	5.4		no	somatic
4	/	/	/	BRCA1DEL Exons16-20; CN = 1	no	somatic
5	/	c.8629G>T, p.(Glu2877Ter)	16		no	somatic
6	/	c.5073dupA, p.(Trp1692Metfs*3)	8.6		no	somatic
7	/	/	/	BRCA1DEL entire gene; CN = 1	no	somatic
8	c.331G>T, p.(Glu111Ter)	/	50.2		no	somatic
9	/	/	/	BRCA1DEL entire gene; CN = 1	no	somatic
	c.2269delG, p.Val757PhefsX8	/	59.2		no	somatic
10	/	/	/	BRCA1DEL entire gene; CN = 1	no	somatic
11	/	/	/	BRCA2DEL entire gene; CN = 1	no	somatic
12	c.1687C>T, p.(Gln563*)	/	79.5		no	somatic
	/	/	/	BRCA2DEL entire gene; CN = 1	no	somatic
13	/	/	/	BRCA1DEL entire gene; CN = 1	no	somatic
14	/	/	/	BRCA2DEL entire gene; CN = 1	no	somatic
15	/	/	/	BRCA2DEL entire gene; CN = 1	no	somatic
16	/	/	/	BRCA2DEL entire gene; CN = 1	no	somatic
17	/	/	/	BRCA2DEL entire gene; CN = 1	no	somatic
18	/	/	/	BRCA2DEL entire gene; CN = 1	no	somatic
19	/	/	/	BRCA2DEL entire gene; CN = 1	no	somatic
20	/	/	/	BRCA2DEL entire gene; CN = 1	no	somatic
21	/	/	/	BRCA1DEL entire gene; CN = 1	no	somatic
22	c.2670delG, p.Ser891Profs*2	/	50.5		no	somatic
23	/	c.6611delC, p.(Pro2204Leufs*2)	54.3		no	somatic
	/	/	/	BRCA2DEL entire gene; CN = 1	no	somatic
24	/	/	/	BRCA1DEL entire gene; CN = 1	no	somatic
25	c.3916_3917delTT, p.Leu1306AspfsX23	/	78.9		yes	germline
26	/	c.7975A>G, p.(Arg2659Gly)	80		yes	germline
27	/	c.3847_3848delGT, p.(Val1283LysfsX2)	85		yes	germline
28	/	c.6037A>T, p.(Lys2013Ter)	85		yes	germline
29	c.547+2T>A	/	83.4		yes	germline
30	c.2157_2160delAGAA, p.(Lys719AsnfsX16)	/	92.8		yes	germline
31	c.4357+1delG	/	90		yes	germline
32	c.3607C>T, p.(Arg1203Ter)	/	59,2		yes	germline
33	c.4096+1G>A	/	73.4		yes	germline
34	c.3481_3491delGAAGATACTAG, p.(Glu1161PhefsTer3)	/	91.4		yes	germline
35	c.843_846delCTCA, p.(Ser282TyrX15)	/	83.2		yes	germline
	/	/	/	BRCA1DEL entire gene; CN = 1	no	somatic
36	c.3288_3289delAA, p.(Leu1098SerfsX4)	/	94.6		yes	germline
37	c.5434C>G, p.(Pro1812Ala)	/	63.8		yes	germline
38	/	c.9097dupA, p.(Thr3033AsnfsX11)	Tissue not available		yes	germline
39	c.5017_5019delCAC, p.(His1673del)	/	64.8		yes	germline
40	/	c.5722_5723delCT, p.(Leu1908Argfs*2)	60.3		yes	germline
41	/	c.7180A>T, p.(Arg2394*)	78.7		yes	germline
42	c.3916_3917delTT, p.(Leu1306Aspfs*23)	/	76		yes	germline
43	c.4508C>A, p.(Ser1503*)	/	88.6		yes	germline
44	c.4484G>T, p.(Arg1495Met)	/	73		yes	germline
45	c.2157_2160delAGAA, p.(Lys719Asnfs*16)	/	84.3		yes	germline
46	c.3979C>T, p.(Gln1327*)	/	84.6		yes	germline
47	/	c.9154C>T, p.(Arg3052Trp)	59.3		yes	germline
48	/	/	/	BRCA1DEL Promotor-Exon2; CN = 1	yes	germline
49	/	c.3847_3848delGT, p.(Val1283Lysfs*2)	77.4		yes	germline
	/	/	/	BRCA2DEL entire gene; CN = 1	no	somatic
50	c.3916_3917delTT, p.(Leu1306Aspfs*23)	/	70.2		yes	germline
51	/	c.7558C>T, p.(Arg2520*)	5.8		yes	germline

## Data Availability

Data are available upon reasonable request.

## Supplementary Materials

The following are available online at https://www.mdpi.com/2075-4418/11/3/565/s1, Table S1: Univariate analysis of pre-specified parameters for OS and PFS.
