# Supplementary material for: The Prognostic and Predictive Role of Somatic BRCA Mutations in Ovarian Cancer: Results from a Multicenter Cohort Study
Source: Diagnostics (Basel). 2021 Mar 21;11(3):565. doi: 10.3390/diagnostics11030565 (PMC8003908; doi:10.3390/diagnostics11030565)
Supplement: Supplementary file 1 [file diagnostics-11-00565-s001.pdf]

Supplementary Materials

Table 1.

Univariate analysis of pre-specified parameters for OS and PFS.

	**OS**			**PFS**		
	**P value**			**P value**		
	**wtBRCA**	**gBRCA**	**sBRCA**	**wtBRCA**	**gBRCA**	**sBRCA**
Age ≥50 years	0.005	0.54	0.4	0.001	0.56	0.55
BCFH	0.28	0.63	0.44	0.28	0.87	0.16
OCFH	0.92	0.79	0.67	0.84	0.39	0.14
BCOCFH	0.52	0.63	0.29	0.48	0.69	0.57
FIGO stage 3-4	0.04	0.71	0.33	0.001	0.13	0.73
Histotype Serous	0.03	0.77	0.51	0.25	0.81	0.81
Surgery (no)	0.01	0.001	0.09	0.001	0.75	0.001
Residual (yes)	0.02	0.05	0.67	0.01	0.13	0.35
